# A cost-effectiveness analysis of a South African pregnancy support grant

**DOI:** 10.1371/journal.pgph.0002781

**Published:** 2024-02-08

**Authors:** Aisha Moolla, Winfrida Mdewa, Agnes Erzse, Karen Hofman, Evelyn Thsehla, Susan Goldstein, Ciaran Kohli-Lynch

**Affiliations:** 1 SAMRC/Wits Centre for Health Economics and Decision Science ‐ PRICELESS SA, School of Public Health, Faculty of Health Sciences, University of the Witwatersrand, Johannesburg, South Africa; 2 Department of Preventive Medicine, Northwestern University Feinberg School of Medicine, Chicago, Illinois, United States of America; Duke University, UNITED STATES

## Abstract

Poverty among expectant mothers often results in sub-optimal maternal nutrition and inadequate antenatal care, with negative consequences on child health outcomes. South Africa has a child support grant that is available from birth to those in need. This study aims to determine whether a pregnancy support grant, administered through the extension of the child support grant, would be cost-effective compared to the existing child support grant alone. A cost-utility analysis was performed using a decision-tree model to predict the incremental costs (ZAR) and disability-adjusted life years (DALYs) averted by the pregnancy support grant over a 2-year time horizon. An ingredients-based approach to costing was completed from a governmental perspective. The primary outcome was the incremental cost-effectiveness ratio (ICER). Deterministic and probabilistic sensitivity analyses were performed. The intervention resulted in a cost saving of R13.8 billion ($930 million, 95% CI: ZAR3.91 billion ‐ ZAR23.2 billion/ $1.57 billion ‐ $264 million) and averted 59,000 DALYs (95% CI: -6,400–110,000), indicating that the intervention is highly cost-effective. The primary cost driver was low birthweight requiring neonatal intensive care, with a disaggregated incremental cost of R31,800 ($2,149) per pregnancy. Mortality contributed most significantly to the DALYs accrued in the comparator (0.68 DALYs). The intervention remained the dominant strategy in the sensitivity analyses. The pregnancy support grant is a highly cost-effective solution for supporting expecting mothers and ensuring healthy pregnancies. With its positive impact on child health outcomes, there is a clear imperative for government to implement this grant. By investing in this program, cost savings could be leveraged. The implementation of this grant should be given high priority in public health and social policies.

## Introduction

Many South African women are impoverished during pregnancy. A disproportionate number of pregnancies (approximately 69%) occur in impoverished households, and 35% of pregnant women struggle to purchase food [1]. Their economic status is further diminished due to the increased costs associated with accessing antenatal care (ANC), including transport costs and a lack of affordable child care [1–3]. These factors can negatively impact maternal and child health outcomes [2].

The first 1,000 days of life, between conception and a child’s second birthday, is a crucial period of development. During this time, 80% of brain development occurs [4]. Adequate nutrition and care, during this window, can significantly impact not only the child’s survival, but also their long-term ability to grow, learn, and rise out of poverty, ultimately contributing to society’s health, stability, and prosperity [4]. Since the advent of democracy in 1994, free antenatal and postnatal care has been provided to all pregnant women at public-sector primary care facilities, with eight antenatal visits recommended over 40 gestational weeks [5]. However, the mean number of ANC visits is reported to be 5.90 visits [5], with only 83% of all pregnant women in South Africa accessing at least one ANC visit [6]. Inadequate ANC has been linked to neonatal and infant mortality, low birth weight (LBW), and stunting [7]. Further, maternal nutrition during pregnancy is vital for foetal and placental growth and can impact adverse birth outcomes. Impoverished pregnant women often lack adequate nutrients due to a lack of food diversity [8]. Undernutrition during pregnancy may lead to poor foetal nutrition, which increases the likelihood of adverse birth outcomes, including preterm birth, LBW, intrauterine growth restriction, and cognitive impairment [9]. The potential for the child to thrive in life is, therefore, reduced for impoverished mothers for whom costs associated with being pregnant may perpetuate poverty and its negative consequences for nutrition.

The Child Support Grant (CSG) was introduced in South Africa in 1998, following the end of Apartheid, as a response to the health and development consequences of persistent childhood poverty [10]. The CSG currently provides ZAR460 ($31) per child per month from birth until age 18 to families with an annual income less than ZAR48,000 ($3,244) per parent [11]. Around 13 million children, or 82.5% of those eligible, were enrolled in the program in 2022 [11]. Although the early initiation of the CSG can reduce multidimensional poverty and improve childhood health, education, and nutrition outcomes [10], the grant fails to address poverty experienced during the initial antenatal stages of the first 1,000 days of life. No studies on the cost-effectiveness of the CSG have been performed to date.

Across the globe, low-and-middle-income countries (LMICs), such as India, Bangladesh, Mexico, Nepal, Nigeria, Kenya and Brazil, have tried to address the financial burden placed on pregnant women by providing them with pregnancy support grants (PSGs), conditional cash transfers, voucher schemes, and nutrition support. These interventions have been shown to positively affect healthcare utilisation and maternal nutrition, thus improving neonatal mortality, the preterm birth rate, birth weight, intrauterine growth, childhood growth, and development [12, 13]. These outcomes are likely mediated by improvements in both maternal nutrition and ANC. By providing women with income support, their nutrition often improves [14], which results in fewer children who are small-for-gestational-age (SGA), preterm, LBW, stunted or wasted [9]. Furthermore, pregnant women may forego ANC visits due to its impact on employment. There are increased costs associated with accessing ANC, such as transport costs, a lack of affordable childcare needed when the mother is attending ANC visits, and a loss of wages among women who are paid hourly [1–3]. Thus, providing income support will likely improve ANC and lead to improved outcomes such as reduced infant mortality [15]. These factors illustrate the mechanisms through which the PSG may improve child health outcomes.

Income support interventions for pregnant women in low-income and low-middle-income countries, including Bangladesh, Nigeria, Uganda and Myanmar, have been shown to reduce out-of-pocket costs and improve health outcomes, indicating that they are highly cost-effective [16–19]. Conversely, in high-income countries, these interventions have been found to be too costly [20]. This difference in outcomes between high and low-income counties is likely due to higher benefits observed in poorer populations when provided with financial support [21]. South Africa is considered an upper-middle income country with one of the most unequal countries across the globe. Given that no cost-effectiveness studies have taken place in a similar context, consideration of the cost-effectiveness of a pregnancy grant is essential.

The cost-effectiveness of a PSG has not yet been demonstrated in South Africa. This study aimed to assess the cost-effectiveness, from a governmental perspective, of extending the Child Support Grant into the antenatal period using the existing eligibility criteria. This is compared to the current scenario of grant initiation starting during the postnatal period.

## Materials and methods

### Approach

A cost-utility analysis was performed to determine whether a PSG would be cost-effective when provided to pregnant South African women who meet the criteria to receive a CSG to the value of ZAR460 ($31) per child per month (i.e. an annual income of less than ZAR48,000 ($3,244) per parent, equivalent to a monthly income of less than R4,000 ($270) per parent up to a maximum of two parents). This type of analysis was selected in order to allow policy makers to compare health outcomes of this intervention with others that are under consideration. The analysis was conducted from a governmental perspective, where the Department of Social Development would bear the cost of the proposed grant. An analytical time horizon of the first 1,000 days of life (conception to age two years) was implemented as the costs and benefits associated with the grant largely occur during the period between conception and 24 months of age.

### Intervention and comparator

The intervention evaluated in this study was the PSG (ZAR460 [$31] per month), which would be given to South African pregnant women, including teenagers, in addition to the existing post-natal CSG. It was assumed that the grant would be collected one month after the first ANC visit, which is reported to be at a median gestation of four months [22]. Hence, the PSG is assumed to begin from month five of gestation to month nine at a value of ZAR460 ($31) per month, totalling ZAR2,300 ($155). The comparator was the current CSG alone, valued at ZAR460 ($31) per month, from the first month of life, with no antenatal financial support, making the total cost of the grant ZAR R11,040 ($744).

### Decision tree model

As in previous cost-utility analyses of pregnancy support initiatives [16, 17], an Excel-based decision tree model predicted pregnancy-related health and cost outcomes in a cohort of pregnant South African women. The choice of modelled pregnancy-related events and their probabilities were derived from a review of the available literature, focusing on trials and systematic reviews, and was guided by consultation with clinical experts.

**Fig 1:**
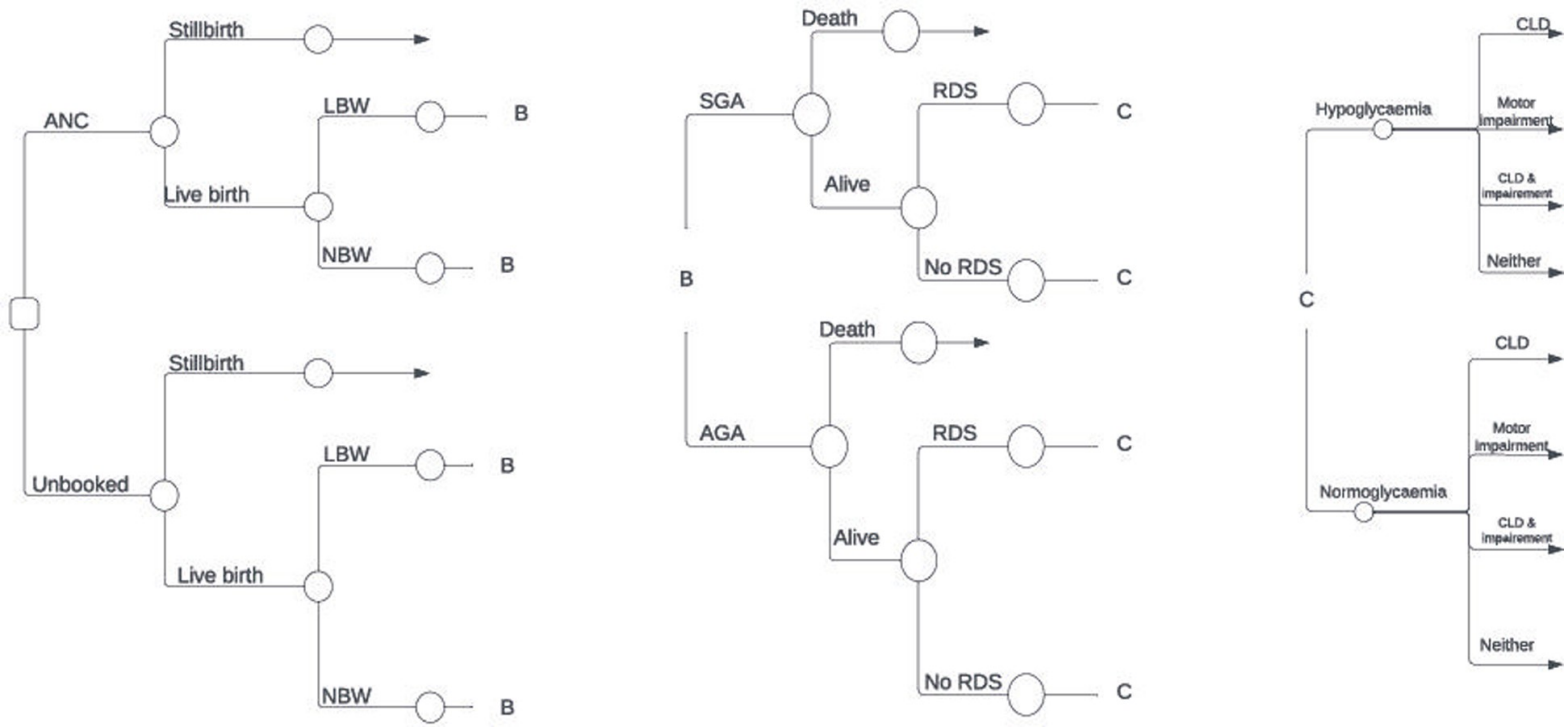
Decision tree model structure. Abbreviations: AGA, appropriate-for-gestational age; ANC, antenatal care; CLD, chronic lung disease; LBW, low birth weight; NBW, normal birth weight; RDS, respiratory distress syndrome; SGA, small-for-gestational age.

Fig 1 shows the structure of the model. Modelled pregnancy-related events included antenatal first visit coverage, stillbirth and infant death, preterm birth, LBW, and SGA, as these parameters were reported to improve when pregnant women received income support. The primary complications of these pregnancy-related events were included in the model based on expert consultation. These were neonatal respiratory distress syndrome (RDS), chronic lung disease, neonatal hypoglycaemia, and motor impairment. Neonates born before 37 weeks of gestation were considered preterm [23], and infants were considered LBW if their birth weight was less than 2,500g [24].

### Model inputs

Table 1 shows the base case probabilities, effects of the grant, assumptions, disability weights (DWs), DALYs, and total costs. Based on the recommendation of the South African Pharmacoeconomic Guidelines [25], a 5% discount rate was applied to costs and benefits.

**Table 1:** Model inputs and deterministic parameter values.

Parameter	Base case	Low value	High value	Distribution for PSA	Source
Total pregnancies	1,337,632	n/a	n/a	n/a	[6]
Proportion of pregnancies among non-citizens	25%	n/a	n/a	n/a	[6]
Pregnancies (South African citizens)	1,003,224	n/a	n/a	n/a	Calculation
Proportion of pregnancies eligible for the CSG	44%	n/a	n/a	n/a	[1]
Number of pregnancies eligible for the CSG	441,418.48	n/a	n/a	n/a	Calculation
Uptake of the CGS (assumed for PSG)	82.6%	n/a	n/a	n/a	[26]
Number of pregnant mothers that take up PSG if eligible	97,754.13	n/a	n/a	n/a	Calculation
Probabilities					
Antenatal first visit coverage	0.83	0.69	1.00	Beta	[6]
Live birth following ANC	0.98	0.49	1.00	Beta	[27]
Live birth in those without ANC	0.97	0.48	1.00	Beta	[27]
Preterm birth following ANC	0.17	0.09	0.26	Beta	[27]
Preterm birth in those without ANC	0.17	0.09	0.26	Beta	[27]
LBW among preterm infants	0.68	0.36	1.00	Beta	[28]
LBW among term infants	0.34	0.17	0.52	Beta	[28]
SGA among preterm LBW infants	0.44	0.22	0.67	Beta	[29]
SGA among preterm normal-weight infants	0.00	n/a	n/a	Beta	[29]
SGA among term LBW infants	1.00	0.50	1.50	Beta	[29]
SGA among term normal-weight infants	0.19	0.10	0.29	Beta	[29]
Death among preterm SGA infants	0.08	0.04	0.13	Beta	[29]
Death among term SGA infants	0.01	0.01	0.02	Beta	[29]
Death among preterm AGA infants	0.04	0.02	0.05	Beta	[29]
Death among term AGA infants	0.006	0.00	0.01	Beta	[29]
RDS among preterm infants	0.45	0.23	0.68	Beta	[23, 30]
RDS among term infants	0.00	n/a	n/a	Beta	[30]
CLD among those with RDS	0.36	0.18	0.54	Beta	[31]
CLD among those without RDS	0.00	n/a	n/a	Beta	[31]
Hypoglycaemia among preterm SGA infants	0.22	0.11	0.32	Beta	[32]
Hypoglycaemia among term SGA infants	0.26	0.13	0.39	Beta	[33]
Hypoglycaemia among preterm AGA infants	0.15	0.08	0.23	Beta	[34]
Hypoglycaemia among term AGA infants	0.00	n/a	n/a	Beta	[35]
Motor impairment in infants with hypoglycaemia	0.051	0.03	0.08	Beta	[36]
Motor impairment in infants without hypoglycaemia	0.033	0.02	0.05	Beta	[36]
Effect Sizes, Risk Ratio					
Increase in antenatal first-visit coverage with a PSG	0.10	0.05	0.15	Log-Normal	[37]
Reduction in preterm birth with a PSG	0.76	0.69	0.84	Log-Normal	[38]
Reduction in LBW with a PSG	0.71	0.63	0.82	Log-Normal	[38]
Reduction in SGA with a PSG	0.90	0.82	0.99	Log-Normal	[38]
Reduction in infant death with a PSG	0.09	0.04	0.13	Log-Normal	[39]
DW and DALY inputs					
CLD DW	0.133	0.086	0.192	Beta	[40]
Years lived with CLD (years)	65.5	n/a	n/a	n/a	[41]
Life expectancy at age of premature death (CLD) (years	65.5	n/a	n/a	n/a	[41]
Motor impairment DW	0.061	0.04	0.089	Beta	[40]
Years lived with motor impairment (years)	30	n/a	n/a	n/a	[42]
Life expectancy at age of premature death (motor impairment) (years)	40.89	n/a	n/a	n/a	[42]
Terminal node DALYs					
Death	19.18	n/a	n/a	n/a	Calculation
Healthy	0.00	n/a	n/a	n/a	Calculation
CLD	0.53	0,27	0,80	n/a	Calculation
Motor impairment	17.27	8,64	25,91	n/a	Calculation
Costs per pregnancy, ZAR (US$)					
Cost of the PSG	R2,300.00 ($155.45)	R1,150.00 ($77.73)	R3,450.00 ($233.18)	Gamma	[43, 44]
Cost of the CSG	R11,040.00 ($746.17)	R5,520.00 ($373.09)	R11,040.00 ($746.17)	Gamma	[43, 44]
Cost of ANC visit	R177.00 ($11.96)	R88.50 ($5.98)	R265.50 ($17.94)	Gamma	[43, 44]
Cost of care for preterm infants	R577.90 ($39.06)	R288.95 ($19.53)	R866.85 ($58.59)	Gamma	[43, 44]
Cost of care for LBW infant	R254,701.56 ($17,214.80)	R99,325.88 ($6,713.25)	R297,977.65 ($20,139.75)	Gamma	[43, 44]
Cost of care for NBW infant	R1,255.20 ($84.84)	R1,549.44 ($104.72)	R4,648.32 ($314.17)	Gamma	[43, 44]
Cost of care for stillbirth infant	R1,792.00 ($121.12)	R896.00 ($60.56)	R2,688.00 ($181.68)	Gamma	[43, 44]
Cost of care for RDS infant	R30,595.61 ($2,067.90)	R15,297.81 ($1,033.95)	R45,893.42 ($3,101.85)	Gamma	[43, 44]
Cost of care for hypoglycaemic infant	R2,936.26 ($198.46)	R1,468.13 ($99.23)	R4,404.39 ($297.68)	Gamma	[43, 44]
Cost of care for CLD infant	R42,582.51 ($2,878.07)	R21,291.26 ($1,439.04)	R63,873.77 ($4,317.11)	Gamma	[43, 44]
Cost of care for children with motor impairment	R3,819.00 ($258.12)	R1,909.50 ($129.06)	R5,728.50 ($387.18)	Gamma	[43, 44]
Cost of follow-up at a hospital	R907.00 ($61.30)	R453.50 ($30.65)	R1,360.50 ($91.95)	Gamma	[43, 44]
Cost of follow-up at a clinic	R3,363.00 ($227.30)	R1,681.50 ($113.65)	R5,044.50 ($340.95)	Gamma	[43, 44]

Abbreviations: AGA, appropriate-for-gestational age; ANC, antenatal care; CLD, chronic lung disease; CSG, child support grant; DW, disability weight; LBW, low birth weight; NBW, normal birth weight; PSG, pregnancy support grant; RDS, respiratory distress syndrome; SGA, small-for-gestational-age.

n/a–not included in univariate sensitivity analysis

### Costs and benefits

An ingredients-based approach was used to determine costs. Economic costs of the grants (PSG and CSG) and direct medical costs associated with each pregnancy-related event were considered. Costs were presented and expressed in ZAR (2021) or US dollars with an annual average conversion rate of ZAR14.7955 to the dollar (2021). The identification of resource utilisation was determined by a review of local neonatal treatment guidelines [45] and through expert consultation. Unit costs were obtained from the South African uniform fee schedule for 2021 [44] and the 2021 Master Procurement Catalogue [43] for medication. The benefits associated with the intervention and comparator were expressed using DALYs, a standardised metric often used in analyses in LMICs [46].

### Analysis

The primary model outcomes were DALYs averted, healthcare costs, and the incremental net monetary benefit (INMB). The latter value quantifies the health benefits associated with the intervention in monetary terms, accounting for any costs incurred [47]. A positive INMB indicates that an intervention is cost-effective. Results were presented on a cost-effectiveness plane. A country-specific threshold of ZAR38,500 ($2,602) per DALY averted was used [48]. The disaggregated costs and DALYs were estimated to identify drivers of incremental costs and DALYs.

### Sensitivity analysis

A univariate sensitivity analysis was performed whereby base case parameters were varied independently while all other parameters were held constant. Parameters were varied by the upper and lower bounds of their 95% confidence interval (CI) when available or by 50% of the base case otherwise. Changes in model outcomes were then recorded, and the results were presented using a tornado diagram. A probabilistic sensitivity analysis was also performed, the results of which were presented in a cost-effectiveness acceptability curve [49]. The mean and 95% uncertainty interval were calculated for model outcomes from 1,000 probabilistic simulations in which model inputs were sampled from Table 1 distributions.

### Ethics approval

This study received an ethics waiver from the Human Ethics Committee at the University of the Witwatersrand (W-CBP-210910-01) due to the use of secondary data and the exclusion of study participants. No participant consent was required. This study followed the Consolidated Health Economic Evaluation Reporting Standards reporting guideline (S1 Table) [50] and conforms to the principles embodied in the Declaration of Helsinki.

## Results

### Cost-effectiveness

Table 2 shows that the total costs were approximately ZAR40.2 billion ($2.7 billion) and ZAR54 billion ($3.7 billion) for the intervention and the comparator, respectively. The intervention, therefore, resulted in a cost saving of ZAR13.8 billion ($930 million, 95% CI: ZAR3.91 billion ‐ ZAR23.2 billion [$1.57 billion ‐ $264 million]) as a result of reduced neonatal complications that drive up costs. Similarly, Table 2 shows the expected DALYs attributed to the intervention and comparator. Fewer DALYs were accrued in the intervention, resulting in 59,000 (95% CI: -6,400–110,000) DALYs averted by the intervention. This is due to a reduction in early neonatal complications that often result in longer-term complications and death.

**Table 2:** Incremental base case costs and DALYs in the intervention and comparator arms.

	Costs (ZAR [US$ 2021]) (95% CI)	DALYs (95% CI)
**PSG**	ZAR40.2 billion ($2.72 billion) (95% CI: ZAR28.9 billion ‐ ZAR54.1 billion [$1.96 billion-$3.66 billion])	519 thousand (363 thousand ‐ 694 thousand)
**CSG**	ZAR54 billion ($3.65 billion) (95% CI: ZAR37.8 billion ‐ ZAR73.8 billion [$2.56 billion-$4.99 billion])	578 thousand (418 thousand ‐ 752 thousand)
**Incremental**	-ZAR13.8 billion (-$930 million) (95% CI: -ZAR3.91 billion ‐ -ZAR23.2 billion [-$1.57 billion-	59 thousand (-6,4 thousand ‐ 110 thousand)
	-$264 million])	
**INMB**	ZAR16 billion ($1 billion, 95% CI: ZAR8 billion-ZAR23 billion [$551 million ‐ $1.5 billion])	

Abbreviations: DALYs, disability-adjusted life years.

Based on the abovementioned findings, the PSG was cost-saving (i.e., increased health with reduced costs) and is therefore highly cost-effective. The INMB of the PSG was estimated to be ZAR16 billion ($1 billion, 95% CI: ZAR8 billion-ZAR23 billion [$551 million ‐ $1.5 billion]).

### Disaggregated outcomes

The disaggregated expected costs for each pregnancy-related event are included in S2 Table. The primary cost driver was LBW, which incorporates the cost of neonatal intensive care. Given that the intervention prevents LBW, it results in a substantial reduction in costs.

S3 Table shows the disaggregated DALYs derived from each terminal pregnancy-related event in the model. Death, in the form of a stillbirth or infant death, contributed most significantly to the DALYs accrued. By preventing newborn deaths, which contribute considerably to the years of life lost owing to early mortality, the intervention significantly reduces DALYs. In contrast, more DALYs are attributed to the intervention for motor impairment as a result of more children surviving in the intervention group, leading to a greater proportion of children with motor impairment.

### Sensitivity analysis

**Fig 2:**
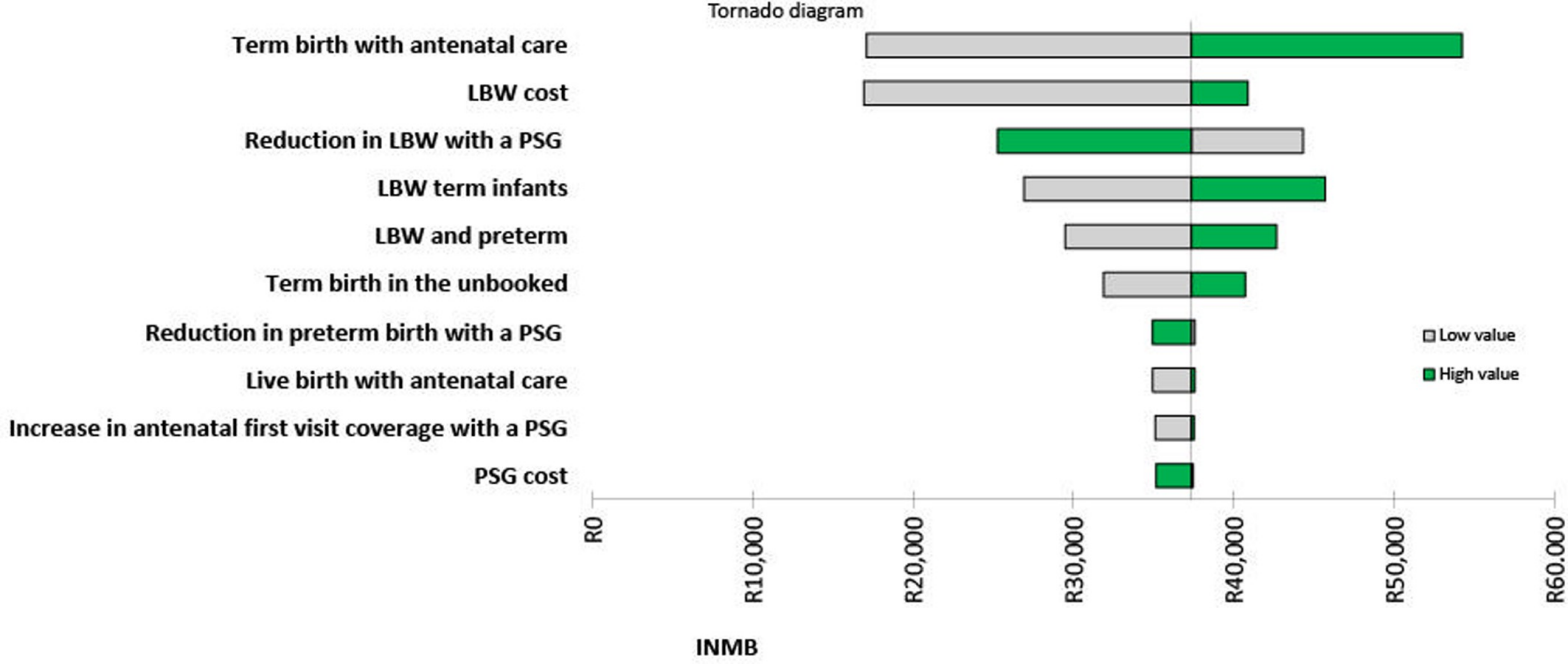
Tornado diagram for top 10 parameters. Abbreviations: LBW, low birth weight; PSG, pregnancy support grant.

In the deterministic sensitivity analysis, the greatest variation in the ICER and INMB was obtained when varying the probability of a term birth with ANC. This was due to the compounded effect of the pregnancy grant and ANC on neonatal health outcomes. Fig 2 presents the results of the univariate sensitivity analysis in the form of a tornado diagram for the ten parameters with the greatest effect on the INMB. It indicates that varying individual parameters still resulted in a positive INMB, showing that the intervention remains cost-effective.

**Fig 3:**
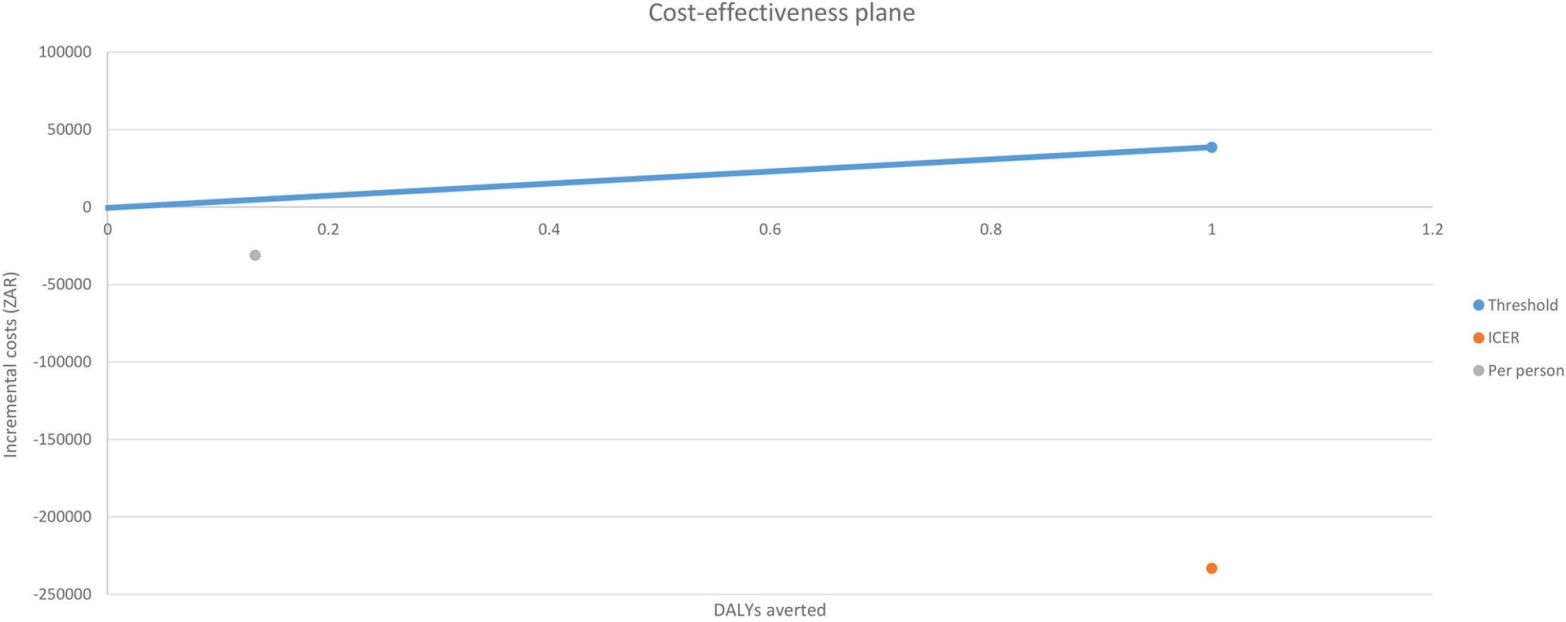
Cost-effectiveness plane. Abbreviations: DALYs, disability-adjusted life years; ICER, incremental cost-effectiveness ratio.

Fig 3 shows the results of the probabilistic analysis, presented on a cost-effectiveness plane. When running the Monte Carlo simulation, 99.5% of iterations were considered cost-effective, indicating a low degree of uncertainty in model outcomes.

**Fig 4:**
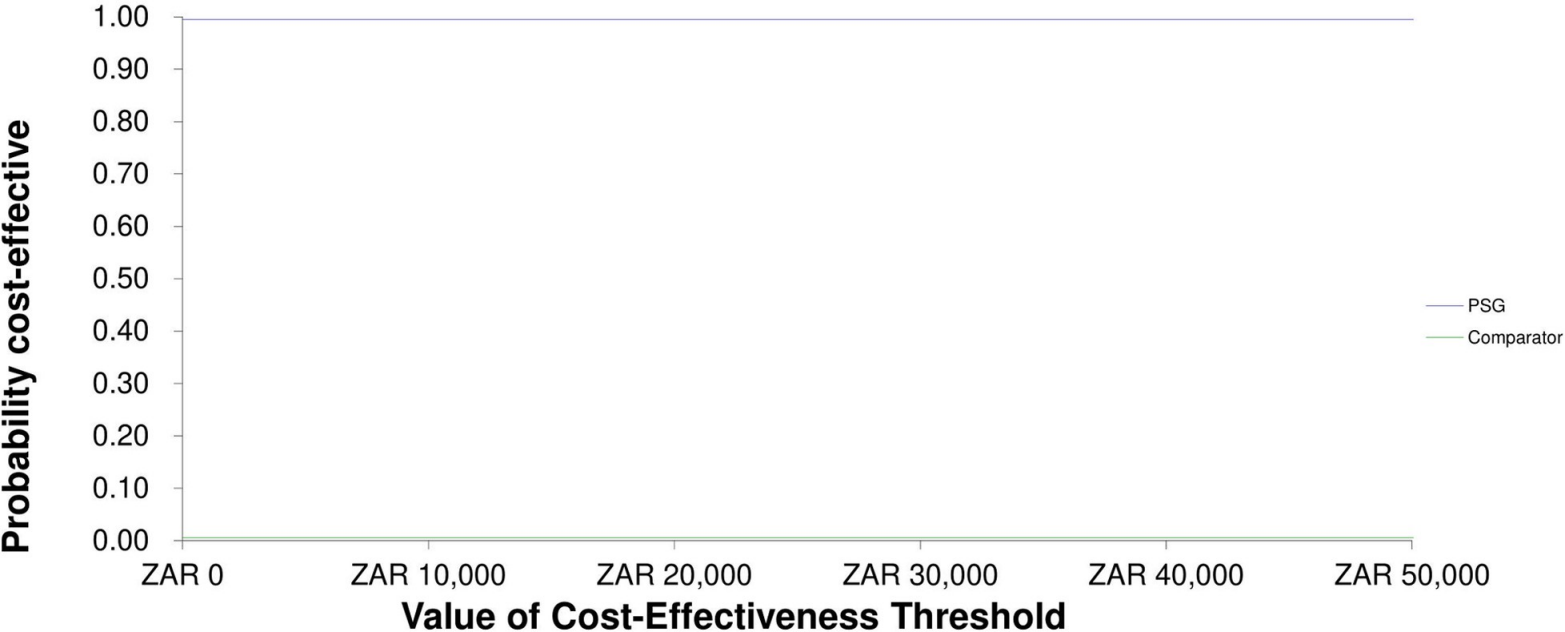
Cost-effectiveness acceptability curve. Abbreviations: PSG, pregnancy support grant.

Fig 4 shows the cost-effectiveness acceptability curve. The graph shows that in most iterations of the simulation, the PSG was cost saving, regardless of a change in cost-effectiveness threshold.

## Discussion

This study aimed to determine whether extending the South African CSG into the antenatal period would be cost-effective. We found that the PSG would be highly cost-effective due to an improvement in child health outcomes, resulting in a net reduction in healthcare costs and DALYs. Around 1.3 million pregnancies occur annually and this grant could benefit 400,000 pregnant women [1, 26].

Our results are similar to studies in other LMICs, which showed that conditional cash transfers and voucher schemes are cost-effective as a form of support during pregnancy [16–18]. Although the effectiveness and cost-effectiveness of these interventions are evident, it is important to determine which population sub-groups would benefit most from financial support during pregnancy. One study showed that the frequency of ANC visits was lower among impoverished women when compared to those without financial constraints [51]. Future research could investigate if this avoidable variation in women’s ability to access critical services may be mitigated by a PSG, which increases the likelihood of recipients accessing ANC visits [37]. Besides limiting access to ANC services, poverty among pregnant women has also been linked to poor neonatal outcomes, including mortality [52]. Neonatal mortality is of particular concern in rural areas, where mothers’ access to adequate and timely care is more limited than in urban settings [52, 53]. These factors suggest that the PSG may be most beneficial to the poor, particularly those in rural areas, in cases where financial constraints prevent them from accessing ANC services. This demonstrates the potential benefits of the PSG with regards to equity.

Beyond the impacts on health and nutrition, the potential perverse incentives of the grant need to be considered. Oyenubi et al. reported that the South African CSG had increased the number of subsequent pregnancies when comparing women who do and do not receive the grant over a 10-year period [54]. This is in contrast to various cohort studies that showed no association between the CSG and subsequent pregnancy [55, 56], with results indicating an inverse relationship among teenage mothers [56]. The variability in results appears to occur due to different outcomes among age groups. Although women between 20 and 58 years were found to have a significantly greater number of children when receiving a CSG, the same effect was not observed among teenage mothers [57]. These findings indicate the need to carefully consider the method used to distribute the grant.

Furthermore, implementation of the PSG should be feasible if incorporated into the existing CSG programme, given the existing infrastructure [13]. By providing pregnant women with an unconditional grant in the form of cash, nutrition and ANC are likely to improve while maintaining client autonomy and reducing the administrative burden of the grant [13]. It is believed that the decline in malnutrition and mortality resulting from the PSG will lead to an increase in the number of years of schooling and future earnings [58]. It should also be noted that this intervention may result in children who may have otherwise passed away now surviving with potential disabilities. Therefore, it is imperative that other sectors collaborate to ensure that interventions are implemented to address the needs of these children throughout their entire life course.

The funding of the PSG is a significant consideration given the multi-sector impact. The implementation of upstream policies and programs outside of the health sector is vital in addressing the social determinants of health, particularly with the proposed implementation of the South African National Health Insurance [59]. It is suggested that the Department of Social Development assume the financial responsibility of the grant, as this department is also responsible for funding the CSG [60]. However, investment into these interventions may require investments across multiple sectors. For example, the health sector alone may underestimate the investment value of a PSG, particularly when non-health benefits such as cognitive outcomes are not considered [61]. In reverse, other sectors may fail to consider the health benefits of the intervention due to the focus on their own departmental objectives, consequently underestimating the intervention’s true value [61]. Although cost savings produced by the PSG in this model will only be accrued by the Department of Health in the short term, there are significant long-term benefits outside the health sector. LBW and premature births result in substantial neurodevelopmental morbidity, which in turn affects cognitive development and school achievement [62]. Additionally, the percentage of children with special educational needs rises steadily with decreasing birth weight [63]. These outcomes increase special education costs in the short term and affect children’s earning potential in the long run. A study conducted in high-income countries found that each standard deviation increase in birth weight was associated with a 2.75% increase in annual earnings [64]. In addition, low school performance due to cognitive challenges could also affect future earnings through delayed entry into the labour force or fewer years of learning [65]. Delayed entry into the labour force results in delaying earnings, thereby reducing the discounted future stream of earnings. There is evidence that verifies that each additional year of schooling completed leads to increased earnings on average [66]. This highlights the need for inter-sectoral collaboration in order to improve overall health, particularly when interventions outside of the health sector achieve health gains more efficiently than the marginal productivity of the health sector [61]. This issue may be dealt with using a co-financing approach [61].

### Limitations and strengths

The limitations of this article include the exclusion of productivity losses associated with adverse pregnancy outcomes and a relatively short time horizon. This may result in an underestimation of the cost savings and benefits accrued over the life course. However, the study strengthens the evidence base of the cost-effectiveness of a PSG. Firstly, it is the first economic evaluation to examine the effect of a PSG, specifically in Sub-Saharan Africa, and it fills a gap in the literature by analysing the impact of a grant in this context. Secondly, the study inputs were primarily sourced from South Africa, making it relevant to the local context. Finally, the decision tree modelling employed in the study included additional health states and complications, which may have provided a more accurate assessment of the benefits and reductions in costs associated with the PSG compared to previous studies in LMICs.

Further research is required to evaluate the cost-effectiveness and budget impact of the intervention from a societal perspective over a lifetime horizon, taking into consideration the inter-generational benefits of the grant. Stratifying the model across different population and socioeconomic groups is recommended in order to determine which groups would benefit most from the PSG in order to ensure that the policy is equitable. Additionally, methodologies need to be developed to assess the cost-effectiveness of multi-sectoral programmes such as the PSG.

## Conclusion

The existing child support grant, while beneficial for alleviating poverty-related child outcomes, fails to address antenatal poverty. Introducing a PSG would result in significant cost savings and improved health outcomes for infants. While the PSG primarily focuses on health outcomes in this study, it is likely to have benefits that extend beyond the first 1,000 days and improve the overall well-being of the child over the life course. The benefits will extend to non-health domains such as educational outcomes and potential earnings of the child later in life. This study demonstrates the benefit of an inter-sectoral response to poverty among pregnant women and indicates that a PSG is a potential best-buy for governmental consideration.

## Supporting information

S1 Table CHEERS checklist. (DOCX)

S2 Table Disaggregated costs for each pregnancy-related event. Abbreviations: ANC, antenatal care; CLD, chronic lung disease; CSG, child support grant; LBW, low birth weight; NBW, normal birth weight; PSG, pregnancy support grant; RDS, respiratory distress syndrome.
(DOCX)

S3 Table Disaggregated DALYs for each terminal pregnancy-related event. Abbreviations: CLD, chronic lung disease; DALYs, disability-adjusted life years.
(DOCX)
