# Supplementary material for: A cost-effectiveness analysis of a South African pregnancy support grant
Source: PLOS Glob Public Health. 2024 Feb 8;4(2):e0002781. doi: 10.1371/journal.pgph.0002781 (PMC10852248; doi:10.1371/journal.pgph.0002781)
Supplement: S2 Table — Abbreviations: ANC, antenatal care; CLD, chronic lung disease; CSG, child support grant; LBW, low birth weight; NBW, normal birth weight; PSG, pregnancy support grant; RDS, respiratory distress syndrome. (DOCX) [file pgph.0002781.s002.docx]

# **S2 Table.** **Disaggregated costs for each pregnancy-related event**

| **Cost category** | **Intervention (costs)** | **Comparator (costs)** | **Cost difference** |
| --- | --- | --- | --- |
| **PSG** | R2,300.00 ($155.45) | - | - |
| **CSG** | R10,714.65 ($724.18) | R10,650.87 ($719.87) | -R63.78 (-$4.31) |
| **ANC** | R161.94 ($10.95) | R147.09 ($9.94) | -R14.86 (-$1.00) |
| **Preterm birth** | R75.37 ($5.09) | R98.97 ($6.69) | R23.60 ($1.60) |
| **LBW** | R69,631.76 ($4,706.28) | R101,431.64 ($6,855.57) | R31,799.87 ($2,149.29) |
| **NBW** | R889.82 ($60.14) | R731.51 ($49.44) | -R158.31 (-$10.70) |
| **Stillbirth** | R31.74 ($2.15) | R34.01 ($2.30) | R2.28 ($0.15) |
| **Infant death** | R21.07 ($1.42) | R29.15 ($1.97) | R8.08 ($0.55) |
| **RDS** | R1,724.28 ($116.54) | R2,240.14 ($151.41) | R515.86 ($34.87) |
| **Hypoglycaemia** | R285.19 ($19.28) | R368.48 ($24.90) | R83.29 ($5.63) |
| **CLD** | R2,015.25 ($136.21) | R3,361.77 ($227.22) | R1,346.52 ($91.01) |
| **Motor impairment** | R152.11 ($10.28) | R148.17 ($10.01) | -R3.94 (-$0.27) |
| **CLD and motor impairment** | R80.34 ($5.43) | R135.21 ($9.14) | R54.87 ($3.71) |
| **Healthy** | R2,994.11 ($202.37) | R2,869.45 ($193.94) | -R124.65 (-$8.42) |

Abbreviations: ANC, antenatal care; CLD, chronic lung disease; CSG, child support grant; LBW, low birth weight; NBW, normal birth weight; PSG, pregnancy support grant; RDS, respiratory distress syndrome.
