# Supplementary material for: A cost-effectiveness analysis of a South African pregnancy support grant
Source: PLOS Glob Public Health. 2024 Feb 8;4(2):e0002781. doi: 10.1371/journal.pgph.0002781 (PMC10852248; doi:10.1371/journal.pgph.0002781)
Supplement: S3 Table — Abbreviations: CLD, chronic lung disease; DALYs, disability-adjusted life years. (DOCX) [file pgph.0002781.s003.docx]

# **S3 Table. Disaggregated DALYs for each terminal pregnancy-related event**

| **Terminal pregnancy-related event** | **Intervention (DALYs)** | **Comparator (DALYs)** | **Difference in DALYS** |
| --- | --- | --- | --- |
| **Death (stillbirth and infant death)** | 0.57 | 0.68 | 0.11 |
| **CLD** | 0.02 | 0.04 | 0.02 |
| **Motor impairment** | 0.56 | 0.54 | -0.01 |
| **CLD and motor impairment** | 0.03 | 0.05 | 0.02 |
| **Healthy** | 0.00 | 0.00 | 0.00 |

Abbreviations: CLD, chronic lung disease; DALYs, disability-adjusted life years.
